
JOURNAL INFORMATION
==============================
NLM Title Abbreviation: Chic Med J
NLM Journal ID: 0027576

The Chicago Medical Journal

Publisher: Biomedical Journal Digitization Project

ARTICLE INFORMATION
==============================
PMCID: PMC9721728
PMID: 37411182
Article version: 1
Subjects: Extracts

Strychnine Poisoning Relieved with Camphor

Print publication date: 1858 Dec
Volume: 15
Issue: 12
First page: 668
Last page: 668
License: This work is free of known copyright restrictions. For more information, please see PMC Back Issue Digitization.
License URL: https://creativecommons.org/publicdomain/mark/1.0/

==============================

STRYCHNINE POISONING RELIEVED WITH CAMPHOR.

In the November, 1858, number of the *Ohio Med. and Surg. Journal*, Dr. Denig reports a case of poisoning by strychnine, which he treated with chloroform and pulv. camphor, the result being favorable. When called to see the patient she was in tetanic convulsions, and, as he satisfactorily ascertained, had taken, for purposes of self-destruction, about *one grain* of strychnine. Dr. D. administered five grs. pulv. camph. every ten minutes, administering chloroform in the meantime, for about half an hour, at the end of which time the spasms ceased to return.

---

FRESH · VACCINE VIRUS,

**Constantly on hand and for Sale, by**

J. H. REED & CO., WHOLESALE DRUGGISTS, 144 & 146 LAKE STREET.

---

GALE BROTHERS,

APOTHECARIES AND CHEMISTS

**202 Randolph Street, Chicago.**

We are prepared to furnish Physicians with Medicines and Chemicals of the most reliable qualities, selected and prepared expressly for our own dispensing department.

New and rare Chemicals and Pharmaceutical Preparations furnished in quantities to suit Practitioners, to whom we recommend an examination of our stock.

---

ARTIFICIAL EYES!

We have a large assortment of *Artificial Human Eyes*, of the most approved manufacture.

**GALE BROTHERS,**

APOTHECARIES AND PHARMACEUTISTS,

202 Randolph St., Chicago.

---

TO THE MEDICAL PROFESSION.

The subscribers would call the attention of physicians to the annexed list of Fluid Extracts, which we have been induced to prepare, from the difficulty of obtaining such preparations of a reliable character, and to obviate the great inconvenience of being dependent on distant manufacturers for articles of every day use by physicians.
